# Instruction-Level Power Side-Channel Leakage Evaluation of Soft-Core CPUs on Shared FPGAs

**DOI:** 10.1007/s41635-023-00135-1

**Published:** 2023-10-04

**Authors:** Ognjen Glamočanin, Shashwat Shrivastava, Jinwei Yao, Nour Ardo, Mathias Payer, Mirjana Stojilović

**Affiliations:** School of Computer and Communication Sciences, EPFL, Route Cantonale, Lausanne, 1015 Vaud Switzerland

**Keywords:** FPGA, Multitenancy, CPU instruction identification, Power side-channel attack

## Abstract

Side-channel disassembly attacks recover CPU instructions from power or electromagnetic side-channel traces measured during code execution. These attacks typically rely on physical access, proximity to the victim device, and high sampling rate measuring instruments. In this work, however, we analyze the CPU instruction-level power side-channel leakage in an environment that lacks physical access or expensive measuring equipment. We show that instruction leakage is present even in a multitenant FPGA scenario, where the victim uses a soft-core CPU, and the adversary deploys on-chip voltage-fluctuation sensors. Unlike previous *remote* power side-channel attacks, which either require a considerable number of victim traces or attack large victim circuits such as machine learning accelerators, we take an evaluator’s point of view and provide an analysis of the instruction-level power side-channel leakage of a small open-source RISC-V soft processor core. To investigate whether the power side-channel traces leak secrets, we profile the victim device and implement various instruction opcode classifiers based on both classical machine learning algorithms used in disassembly attacks, and novel, deep learning approaches. We explore how parameters such as placement, trace averaging, profiling templates, and different FPGA families (including a cloud-scale FPGA) impact the classification accuracy. Despite the limited leakage of the soft-core CPU victim and a reduced accuracy and sampling rate of on-chip sensors, we show that in a worst-case scenario for the evaluator, i.e., an attacker breaching physical separation, we can identify the opcode of executed instructions with an average accuracy as high as 86.46%. Our analysis shows that determining the executed instruction type is not a classification bottleneck, while leakages between instructions of the same type can be challenging for deep learning models to distinguish. We also show that the instruction-level leakage is significantly reduced in a cloud-scale FPGA scenario with higher soft-core CPU frequencies. Nevertheless, our results show that even small circuits, such as soft-core CPUs, leak potentially exploitable information through on-chip power side channels, and users should deploy mitigation techniques against disassembly attacks to protect their proprietary code and data.

## Introduction

Due to the end of Moore’s law and the breakdown of Dennard’s scaling, datacenters are transitioning from homogeneous and processor-dominated systems towards heterogeneous architectures. As a result, today’s datacenters feature not only central processing units (CPUs), but also graphics processing units (GPUs) and special-purpose integrated circuits such as field-programmable gate arrays (FPGAs). FPGAs reached wide deployment in datacenters thanks to their highly parallel architecture, programmability, and energy efficiency [1, 2]. Even though FPGA vendors offer FPGA-based system-on-chips (SoCs) with hardened CPUs [3, 4], cloud providers are exclusively integrating regular FPGAs in their servers because servers are already abundant in high-end server-grade CPUs. Amazon EC2 F1 [1], Azure [2], Baidu [5], and Tencent [6] deploy AMD Virtex or Kintex Ultrascale+ FPGAs, while Alibaba deploys Intel Arria 10 and Agilex FPGAs [7].

In the FPGA-accelerated cloud, highly-parallel tasks are accelerated on FPGAs. At the same time, developers rely on host CPUs for general computation, particularly noniterative and user event-dependent control algorithms, which are significantly easier to implement and maintain in software than in hardware. However, the FPGA-CPU communication incurs high latency, especially for short data transfers [8]. If such delays are of no concern, then the control software can be deployed on a cloud CPU instance; yet, only a limited range of FPGA applications—usually data movement ones—can afford the resulting communication latency. Therefore, in the case of latency-critical control algorithms, system designers resort to using soft-core CPUs as real-time co-processors (e.g., Microblaze [9], Nios [10], PicoRV [11]), which allow tight and customizable integration with FPGA accelerators, and short communication latencies.

Recent efforts focus on extending the multitenancy and resource virtualization from CPUs to FPGAs, to improve the efficiency of datacenter resource provisioning. A specific research focus is achieving spatial or temporal multiplexing of FPGA resources [12–14]. The challenges are numerous, such as partitioning the FPGA resources among multiple users, providing communication protocols between the host virtual machine and the accelerators, and ensuring proper physical and logical isolation between the tenants [15].

Unfortunately, FPGA multitenancy introduces security threats that cannot be mediated by physical or logical isolation between tenants. The reason is the shared power delivery network (PDN), which instigates power side-channel attacks, covert communication [16, 17], and denial-of-service and fault attacks [18, 19]. An attacker does not require physical access to the board, as the fine logic and wiring granularity of FPGAs enables malicious users to craft almost arbitrary hardware, which includes implementing on-chip sensors for measuring the shared power supply voltage fluctuations [20]. Several remote power-analysis attacks have already been demonstrated: a simple-power analysis (SPA) attack on RSA exponentiation [21], correlation power analysis (CPA) attacks against AES (requiring a large number of victim power traces) [22–24], and reverse engineering attacks on neural network accelerators (which occupy a significant portion of the FPGA resources) [25–28].

For an FPGA user, secret information is not limited to their bitstream, the cryptographic key, or neural network accelerator parameters and architecture. If their design contains a soft-core CPU, the code being executed can be proprietary or contain secrets. If an attacker, by observing power side-channel traces during CPU code execution, can determine which instructions are being executed, the confidentiality of the code will be compromised. In embedded applications and smart cards, where adversaries have physical access to the target device to measure power and electromagnetic side-channel leakage, attacks that aim at code recovery are termed side-channel disassembly attacks [29, 30]. Unlike statistical-based power analysis attacks such as CPA, side-channel disassembly attacks are profiling attacks and assume the attacker can record a limited number of victim execution traces.

Our work takes an evaluator’s point of view: we explore to which extent soft-core CPUs leak instruction-level information through the remote power side channel, in cases when an evaluator (or a potential attacker) has no physical access to the device but can deploy on-chip voltage-drop sensors. Unlike traditional side-channel disassembly attacks—where the CPU runs at frequencies orders of magnitude lower than the sampling rate of the oscilloscope—sensors used in remote power analysis attacks have sampling frequencies in the same operating range as soft-core CPUs. Our work analyzes if and under which conditions soft-core CPU instructions contain power side-channel leakage and incentivizes the use of protection methods in multitenant FPGAs. As our leakage evaluation targets, we choose two RISC-V soft-core CPUs using the 32-bit RISC-V base integer instruction set architecture (RV32I), most suitable for lightweight real-time co-processors [31].

To start, we record the side-channel traces corresponding to the execution of every CPU instruction. Then, to investigate whether the traces leak secrets, we train diverse machine learning (ML) classifiers used in previous work and also explore the use of novel deep learning (DL) classifiers to improve the extraction of the power side-channel leakage. The results reveal that, despite the limited accuracy and sampling rate of on-chip sensors compared to oscilloscopes used in disassembly attacks with physical access, the limited leakage compared to previous remote reverse-engineering attacks, and the limited number of victim trace acquisitions compared to statistical-based attacks, instruction-level leakages still exist: we can determine the executed instructions with average accuracy higher than 80%. These results call for proper mitigations to limit power side-channel leakage of soft-core CPUs in shared FPGAs.

We make the following contributions:

- To the best of our knowledge, we present the first analysis of instruction-level leakage of soft-core CPUs in a shared FPGA setting.
- While power side-channel traces recorded by an on-chip FPGA sensor during the execution of one RISC-V soft-core CPU instruction contain limited visually observable leakage, we demonstrate that, in certain conditions, advanced ML techniques can extract sufficient information to identify the opcode of the executed instructions. The maximum average instruction accuracy we achieve on the RV32I instruction set architecture (ISA) is 86.46%.
- Besides evaluating previous side-channel disassembly approaches, we explore new, DL-based instruction classifiers, and experimentally find that they are superior at extracting leakage compared to common ML techniques deployed in previous work, and should be used for future side-channel security evaluations.
- We perform an extensive experimental analysis that compares how different leakage evaluation scenarios, such as the number and placement of sensors, number of templates, and type of templates, affect the instruction-level leakage. We also demonstrate our results on two soft CPU cores and two different FPGA families. In addition to the leakage analysis of the RISC-Y [32] soft-core CPU running at 80 MHz on the Sakura-X board [33], we show results on a cloud-scale, AMD Alveo U200 datacenter accelerator card, using the compact PicoRV [11] soft-core CPU, running at 320 MHz. With our on-chip sensors running at 320 MHz, the side-channel traces have only four sensor samples per CPU clock cycle on Sakura-X, and only one sensor sample per CPU clock cycle on Alveo U200; significantly lower than in traditional side-channel disassembly attacks.
- We provide a detailed discussion of our experimental results and their impact on soft-core CPU leakage evaluation, which we use to motivate appropriate mitigation techniques.

Our work aims to provide a leakage evaluation methodology for soft-core CPUs in remotely accessible scenarios and to benefit future power side-channel disassembly attacks by providing novel DL power trace classification techniques. Therefore, we make all our FPGA designs, associated software, and ML code openly available for the reproducibility of the experiments and the results in this work [34].

## Background

Almost a decade ago, Microsoft pioneered the use of FPGAs in cloud computing. Their Catapult project pilot of 1,632 FPGA-enabled datacenter servers demonstrated a dramatic improvement in Bing search latency, launching the era of FPGA-accelerated cloud computing [35]. Other cloud service providers soon followed. Today, Amazon AWS, Azure, Alibaba, Baidu, and Tencent offer their customers remote access to datacenter FPGAs, to develop, test, and deploy their custom hardware accelerators [1, 5–7].

To remote users, FPGAs are typically exposed through a host CPU virtual machine interface and a *shell-role* use model [15]. The shell is deployed by the cloud service providers and shares the FPGA logic with the users. In addition, it implements platform-specific management tasks: PCIe, direct memory access engine, DRAM controller, and debugging interfaces. The FPGA region reserved for each user is called a role, and users deploy their accelerators within their role. The shell-role separation helps faster accelerator deployment and ensures different privilege levels between the cloud service providers and the external users.

In both academia and industry, increased efforts are being made to extend multitenancy and resource virtualization from CPUs to FPGAs, to enable better management and use of available datacenter resources [12, 15, 36–46]. Multitenancy can be achieved through spatial and temporal multiplexing. Temporal multiplexing separates users in time, ensuring that each tenant gets their own, exclusive instance. In spatial multiplexing, FPGA roles are occupied by potentially different tenants, and consequently, the cloud service providers need to ensure security and privacy to all of them [45, 47, 48].

**Fig. 1:**
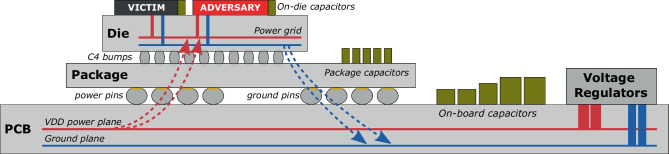
Power delivery network coupling across the board, package, and the FPGA die

Once the shell and the tenants share the FPGA die, they also share the PDN illustrated in Fig. 1. On the printed circuit board (PCB) level, the PDN starts with the primary voltage regulator. The power is then distributed through several levels of voltage regulators if needed, and the power and ground planes. Inside the FPGA, a PDN resembling a dense mesh supplies power to all FPGA logic and routing resources. On all the levels—board, package, chip—the PDN contains resistive, capacitive, and inductive components, some of them intended and some parasitic, which create a medium for voltage fluctuations in one FPGA role to propagate to another. Gnad et al. were the first to demonstrate that a malicious FPGA tenant can, through excessive logic switching, draw too much current and, consequently, reset the host FPGA [18]. Their findings temporarily put on hold the FPGA multitenancy in the cloud and pushed many researchers to investigate new attack surfaces, threat models, and countermeasures [49, 50].

One of such new attack surfaces called *remote power analysis attacks*, was first demonstrated by Zhao and Suh [21] and Schellenberg et al. [22]. Leveraging the fine granularity of reconfigurable logic and routing in FPGAs allowed the designing and implementing of circuits that sense on-chip power supply voltage variations. Unlike traditional power analysis attacks, which require physical access to the victim to measure its power consumption with an oscilloscope [51], using on-chip sensor circuits made these attacks remote, no longer requiring physical access to the device. An example of such a sensing circuit is a simple ring oscillator (RO), composed of an odd number of inverters connected to form a closed chain. Its oscillation frequency depends on the delays of the inverters and routing resources which, in turn, depend on the power supply voltage. Hence, one can also sense the voltage variations by measuring the RO frequency. Another example is a delay-line sensor, also called time-to-digital converter (TDC) [20], which we will discuss in detail in Sect. 4. In a multitenant FPGA setting, an adversary can use such sensors to collect the power side-channel information leaked from a co-located tenant and use it to infer secret information: indeed, on-chip sensors allowed remote attacks on cryptographic circuits, ML accelerators, and other circuits. We summarize the most relevant previous work in Sect. 11.

## Threat Model

Research on the security of multitenant FPGAs follows a well-established threat model of the fault and side-channel attacks on remote shared FPGAs [19, 21, 23, 28, 49, 52–54]. The primary assumption is that at least two users can remotely deploy their designs on the same FPGA instance simultaneously. For security reasons, these remote users are given control over dedicated partial reconfiguration regions, which are logically and physically isolated; thus, the attacker has no direct access or control over the victim or the victim’s deployment. The adversary can deploy voltage fluctuation sensors to record power side-channel traces and send them over the network for remote analysis.

In this work, we assume an evaluator’s point of view: we evaluate the security of a victim that uses a soft processor core in their shared FPGA platform, for example, to configure and control the operation of an accelerator. This work analyzes instruction-level leakage to assess if and under which circumstances soft-core CPUs leak instruction information through the power side channel in shared FPGAs, with the goal of motivating the use of countermeasures.

When evaluating the side-channel security of a device, it is a common practice to consider the worst-case estimates (even if not practically achievable by an attacker), as they quantify the limits of the leakage. For example, in the context of cyber-physical devices, white-box power side-channel leakage evaluation methods leverage proprietary architectural information (unavailable to attackers) to build better power models for power analysis attacks [55]. Removing the plastic cover of a chip to record near-field EM emanations is another example of a common practice in leakage evaluations, even though attackers might not always be able to remove the casing. Consequently, our experiments assume and evaluate various scenarios: from worst-case (a breach of physical and logical separation, no additional noise sources, and averaging of traces) to more realistic scenarios, including physical separation, no averaging, and noise from surrounding instructions and the shell.

**Fig. 2:**
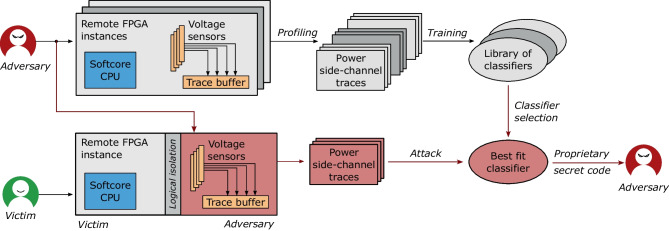
Threat model. The top half illustrates the profiling phase, which results in a library of side-channel instruction classifiers, for a number of FPGA instances and CPU and sensor placements. The bottom half shows the attack

In reality, a hypothetical attacker mounting a profiling attack on soft processor cores would have to perform a procedure similar to the one shown in Fig. 2. To prepare for the attack, an adversary would start by renting an FPGA instance as its only tenant. On this FPGA instance, the adversary would need to calibrate the voltage fluctuation sensors and use them to profile the execution of the CPU instructions for various operating frequencies and several CPU placements. Then, the attacker could train side-channel instruction classifiers. This step would have to be repeated for many FPGA instances, each uniquely identified (e.g., by fingerprinting cloud FPGAs as suggested by Tian et al. [56]).

To perform an exploit using the library of trained classifiers, the attacker would need to rent a shared FPGA instance. Using fingerprinting to identify the shared FPGA instance, the attacker can focus on the subset of the classifiers in the library trained on that particular FPGA instance. Once side-channel traces are obtained, the adversary would need to identify that the co-located user is using a soft-core CPU (and repeat until a victim with a soft-core CPU is identified), using workload classification techniques [54]. Then the attacker could further prune the subset of trained classifiers using the same workload classification techniques—which can distinguish between different soft-core implementations in shared FPGAs—and run the inference. Finally, in addition to the attack procedure, the attacker would need to train models robust to noise from the shell or any other accelerator the victim might be using alongside their soft processor core.

Our aim is to evaluate how and under which circumstances soft-core CPUs leak instruction information in shared FPGAs, we therefore focus on assessing instruction leakage. We refer to related work for FPGA identification and workload classification.

## Experimental Setup

The Sakura-X (Sasebo-GIII) board [33] and the Alveo U200 datacenter accelerator card serve as our target evaluation platforms. Sakura-X is an evaluation board designed for power side-channel analysis and, hence, commonly used in both cryptologic research [57, 58] and research on side-channel attacks on shared FPGAs [22, 27, 59]. Sakura-X has one AMD Kintex-7 FPGA and one AMD Spartan-6 FPGA. The former FPGA is the larger of the two, often referred to as *main* or *target* FPGA, as it hosts the adversary and the victim as two logically isolated FPGA tenants. The second FPGA, often referred to as auxiliary or control FPGA, reduces unwanted noise by implementing the communication protocol between the target FPGA and the host machine [33]. For our evaluation, the Sakura-X architecture increases the already low signal-to-noise ratio (SNR) of soft-core CPUs and helps isolate the instruction-level power side-channel leakage. To evaluate the leakages in a more realistic, cloud-scale FPGA scenario, we use the Alveo U200 datacenter accelerator card. This card contains an AMD UltraScale+ XCU200-2FSGD2104E FPGA, and is commonly used in publicly available cloud FPGA instances [2]. Unlike Sakura-X, Alveo U200 contains a single FPGA consisting of three super-logic regions (SLRs). The shell, containing resources necessary for communicating with the DRAM and host CPU, is instantiated in the middle SLR and physically separated from both the attacker and the victim. The placement of the sensor and the victim CPU varies across experiments, however, in most cases, we physically separate the sensors and the victim soft CPU core to conform with the standard shared FPGA threat scenario described in Sect. 3.

**Fig. 3:**
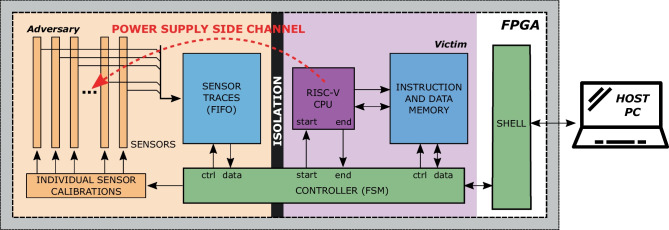
Overview of the experimental setup

Figure 3 gives an overview of the experimental setup for both boards. The target FPGA design contains the victim and the hypothetical attacker logic and has four main components: a soft-core RISC-V processor, the on-chip voltage-drop sensors, the control finite state machine (FSM), and the shell. As discussed in Sect. 1, the primary purpose of using soft-core CPUs is to implement latency-critical control algorithms, especially ones subject to change over time. Therefore, our study assumes the victim uses small soft-core CPUs, common in embedded bare-metal applications [60]. These soft-core CPUs are usually lightweight, with no advanced microarchitectural features such as cashing or speculative execution. They have a low area overhead and can run at high clock frequencies. Their microarchitectural simplicity allows easy and tight integration with FPGA hardware, facilitating low-latency communication. Integrating larger soft-core CPUs would reduce the operating frequency (e.g., Rocketchip can run on a couple of tens of MHz only [61]), increase the area overhead (reducing the available resources for hardware accelerators), and adversely affect the communication latency (as communication would take place through memory mapped interfaces or an operating system).

For the RISC-V soft-core designs, we chose RISCY and PicoRV32, both openly available [11, 32]. Table 1 summarizes the FPGA resource overhead. As a reference, we also show the resource usage of Rocket Chip [61], a larger, more complex soft-core RISC-V implementation. RISCY, used on the Sakura-X board, implements a classic five-stage pipeline and supports the complete RV32I ISA at the cost of a lower operating clock frequency. On Sakura-X, the maximum operating frequency of the RISCY CPU is 100 MHz; however, our system runs it at 80 MHz, to have an integer number of sensor samples per one CPU clock cycle. PicoRV32, used on the Alveo U200 board, has a multicycle CPU microarchitecture designed to minimize resources and maximize the CPU operating frequency. Our system runs PicoRV32 at the maximum operating clock frequency of 320 MHz. In the following subsections, we describe the voltage-drop sensors and the controller in detail.

**Table 1:** Resource utilization of the soft-core CPUs

**CPU**	**FPGA**	**LUT**	**FF**	**BRAM36**	**DSP**
RISCY [32]	Kintex-7	2544	1944	40	0
	XC7K160T-1FBGC				
PicoRV32 [11]	Virtex Ultrascale+	1442	1473	8	0
	XCU200-FSGD2104-2-E				
Rocket Chip [61]	Virtex Ultrascale+	25785	12654	12	15
	XCU200-FSGD2104-2-E				

### FPGA Voltage-Drop Sensors

Commonly deployed FPGA voltage-drop sensors fall into two groups: TDCs and RO-based sensors [49, 62]. They both produce an output in the function of their circuit delay, which is approximately inversely proportional to the supply voltage. Hence, the change in the sensor logic delays indirectly exposes the core voltage fluctuations, caused by the switching activity and power consumption of the victim [21]. The key criterion when choosing between a TDC and an RO-based sensor is the required sensor sampling rate: RO-based sensors cannot be sampled as frequently as TDC sensors. However, RO-based sensors have a smaller footprint and need not be calibrated, unlike TDCs. Good use cases for RO-based sensors are FPGA undervolting-based attacks [53, 63] and covert communication [64]. For side-channel analysis, given the importance of a high sampling rate, TDCs are the preferred solution [22, 23, 28]; they are able to record voltage fluctuations with sampling periods in the nanosecond range [20].

The baseline design of a TDC was proposed by Zick et al. [20]. It consisted of two principal components, (1) one delay line implemented using fast carry chain logic and (2) latches, connected to the output of every delay element in the delay chain. At the input of the carry chain, a high-frequency clock signal was connected; let us refer to it as *input* clock. Another clock signal, the *sampling* clock, of the same frequency but a slightly different phase, was used to capture the propagation depth of the rising edge of the input clock through the delay chain. The propagation depth reflected the changes of the carry logic delay, which were primarily caused by the power supply variations. More recently, TDC sensors have replaced latches with flip flops and used a digital clock manager (DCM) to control the phase delay between the input and the sample clock. Proper selection of the phase shift and the delay line length is critical for correct sensor calibration, i.e., for ensuring that the rising edge is indeed captured and not missed. Since the calibration is a lengthy process of trial and error, in our attack model it must be automated. Hence, we design and implement a TDC with a tunable phase shift mechanism and, as suggested in previous work, we avoid instantiating a DCM primitive to reduce jitter [65].

**Fig. 4:**
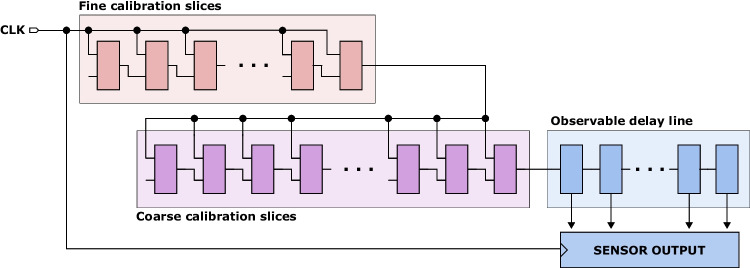
TDC sensor architecture with a tunable phase shift between the clock that enters the observable delay line and the clock that samples the output (i.e., takes a snapshot of the observable delay line). The exact number of slices in our implementation is in Table 2

**Fig. 5:**
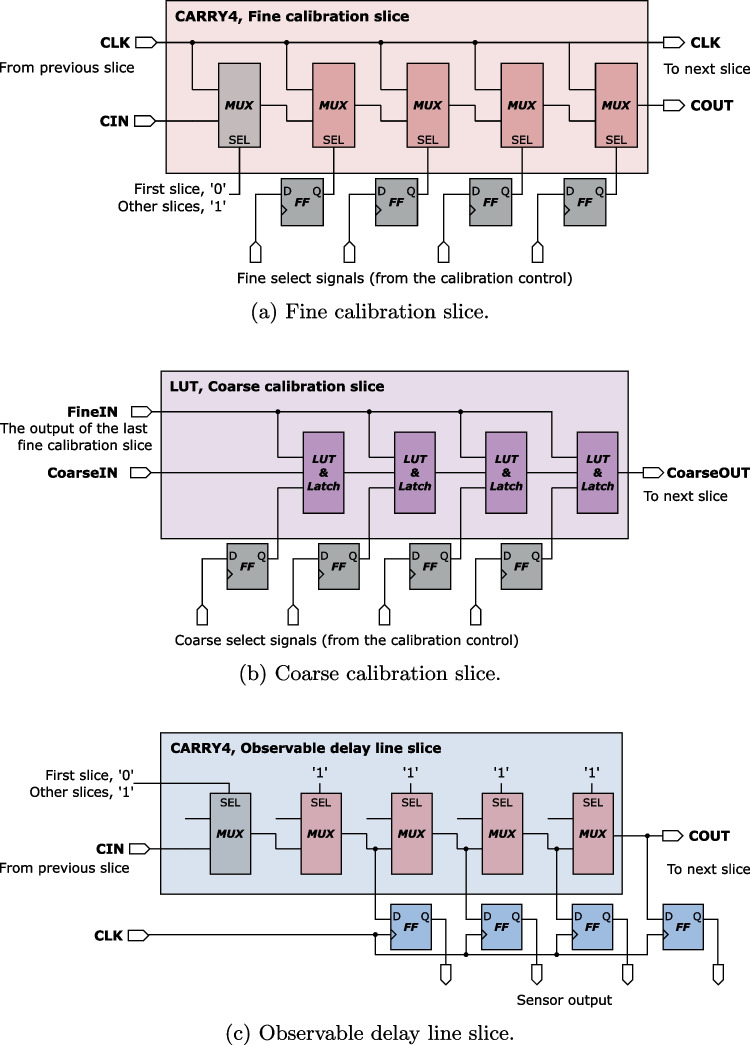
The implementation of each slice in the TDC sensor in Fig. 4, including the calibration and sensor output registers. For space reasons, CARRY4 chain is shown horizontally; in the FPGA design layout, it spans vertically

The design of our TDC is inspired by the implementation of Gnad et al. [16]. Its high-level architecture is shown in Fig. 4. The TDC is composed of fine calibration slices, coarse calibration slices, and an observable delay line, which is periodically sampled and its state saved in the *output register*. The input and the sample clocks are the same. To control the phase shift between the input and the sample clock, *fine* and *coarse* calibration slices are inserted on the input clock path. In the fine calibration slice, as shown in Fig. 5a, calibration inputs control the number of carry chain multiplexers on the clock path. The fine calibration slices are then connected to the coarse calibration slices (Fig. 5b), where the calibration inputs control the number of coarser delay elements on the clock path. In our TDC design, unlike in Gnad et al. [16], coarse delay elements are implemented as LUTs followed by latches, to achieve coarser delay increments. The third and last stage is the *observable delay line* (Fig. 5c). This sensor is considered correctly calibrated when the signal propagating through the chain of delay elements reaches approximately the middle of the observable delay line by the moment it gets captured in the output register.

**Table 2:** Coarse calibration, fine calibration, and observable delay line slices per sensor

**FPGA**	**Fine calibration**	**Coarse calibration**	**Observable line**
Kintex-7	24 slices	8 slices	4 slices
XC7K160T-1FBGC	(= 96 stages)	(= 32 LUTs and Latches)	(= 16 FFs)
Virtex Ultrascale+	12 slices	4 slices	2 slices
XCU200-FSGD2104-2-E	(= 96 stages)	(= 32 LUTs and Latches)	(= 16 FFs)

In this work, TDC sensors have a 16-bit observable delay line. Through experimentation, we found that this length is sufficient to capture the supply voltage variations caused by the CPU operation on both FPGA boards. Table 1 lists the FPGA resources used for our TDC implementation on both boards. The sensor clock frequency was set to 320 MHz on both boards, the highest operating frequency that satisfied timing constraints. Consequently, the sensor captures four samples per one clock cycle of the RISCY CPU running at 80 MHz, and one sample per clock cycle of the PicoRV32 CPU running at 320 MHz.

Previous work has shown that the side-channel information captured by voltage-drop sensors varies with both the absolute location of the sensors as well as their relative position to the victim [66]. It is, therefore, to be expected that an attacker may instantiate more than one power side-channel sensor. The exact number is usually limited by the linearly scaling on-chip memory resources and the data transfer word size. For example, to improve the success of their attack, Gravellier et al. [24] deployed eight sensors on an AMD Artix-7 FPGA. In our experimental setup, we instantiate five TDCs on Sakura-X, and 29 TDCs on Alveo U200, the highest number that fits in a communication message exchanged between the FPGA [33] and the host PC. In Sects. 6 and 7, we will show to what extent having multiple sensors affects the attack efficiency.

### Controller

The controller coordinates the experiments by executing and replying to the commands from the host machine through the shell. It is in charge of initializing the CPU instruction memory with the code to be executed, triggering the execution of the code, and saving the corresponding sensor traces to the on-chip memory. Once the CPU code execution is completed, the controller receives a trigger from the CPU, which initiates the transfer of sensor traces to the host machine. In each message sent from the FPGA to the host, the controller inserts five (Sakura-X) or 29 (Alveo U200) simultaneous sensor readings and the 32-bit word of the corresponding CPU instruction. We replace the default read-only instruction memory of both CPUs with a dual-port block RAM, connecting one memory port to the CPU while exposing the other port to the controller. This temporary change permits the controller to write arbitrary code in the CPU instruction memory before triggering its execution and recording the side-channel traces.

Prior to starting the experiments, the controller calibrates every sensor. The calibration is performed iteratively. First, a test code sequence is loaded to the instruction memory, and the number of elements in the sensor’s initial delay line is set to zero. The code execution is triggered, and the obtained sensor trace is inspected. If no clock transition is observed or the transition is located too close to the two extremes of the observable delay line, the fine and coarse calibration slices are adjusted. This process is repeated until the sensor is calibrated. The calibration settings are then communicated to the host machine for record keeping.

## Instruction Classification

Like all hardware circuits, soft-core CPUs leak information through the power side channel. Various ALU operations, memory accesses, and control-flow changes all impact power consumption differently. In addition, as a combination of fetch, ALU, memory, and program counter operations, instructions also leak information: in the form of unique patterns spread across the time and amplitude domain of the recorded power traces. For example, on the one hand, memory instructions might have high power consumption both in the ALU stage, when the address is computed, and in the later stages of instruction execution, i.e., when the data is read/written to the memory. On the other hand, arithmetic instructions might only have a power consumption peak during the ALU stage.

**Fig. 6:**
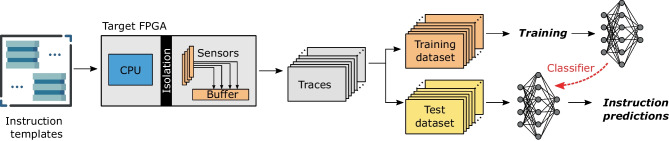
Side-channel instruction leakage evaluation

To analyze the instruction-level power side-channel leakage of soft-core CPUs, we employ an ML-inspired method illustrated in Fig. 6. The key idea behind this approach is that leakage patterns are discovered during ML model training, while the leakage is assessed using the prediction accuracy achieved on templates unseen during training. For this purpose, we first build a large set of template assembly codes for all the target instructions: we generate a set of 10,000 templates for every instruction. Once the templates database is ready, we run the experiments to collect the corresponding power side-channel traces. As leakage evaluators, we reduce the background noise and improve the signal-to-noise ratio by executing each template multiple times and averaging the side-channel traces: 100 times for Sakura-X and 1000 times for Alveo U200. Even though our work represents an instruction-level leakage analysis, averaging is still a commonly used noise reduction approach even in real attack scenarios: for an attack, the victim code is often executed frequently, allowing averaging, while during training, the attacker can execute templates an arbitrary amount of times [67–70]. Finally, to spread out the impact of environmental noise equally across all instruction classes, we record traces in an interleaved fashion: we record a single trace of each class, in a round-robin order, before continuing the acquisition of the next power trace. Subsequently, we prepare the acquired side-channel traces for the training and inference steps. Similar to previous work [67, 71, 72], we partition the final dataset into a training set (for training the instruction classifier) and a test set, for evaluating the instruction classification accuracy and the leakage learned by the models. The following subsections explain the template generation and the training of the side-channel instruction classifiers in greater detail.

### Instruction Template Generation

For our leakage analysis, we create two templating configurations. In the first, denominated as **N**, the target instruction is surrounded by NOP instructions. We use this set of templates to analyze the instruction-level leakage without additional noise from the surrounding instructions. In the second configuration, denominated by **R**, we surround the target instruction with a random instruction before and after. We use the R templating configuration to analyze instruction-level leakage in the presence of other instructions, which represents a more realistic leakage scenario: in practice, the target instruction will be surrounded by a pair of random instructions instead of NOPs.
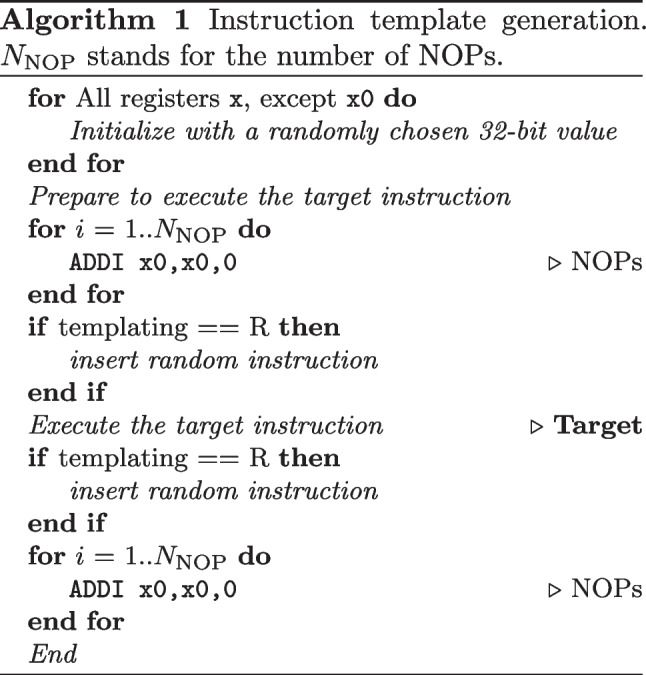


For both templating configurations, we generate 10,000 templates for every instruction from the RV32I ISA, which are listed in Table 3. The process of template generation is detailed in Algorithm 1. The first step is the initialization of x registers with random values. Then, if needed, we insert additional preparation instructions (e.g., to initialize the contents of a memory location for the load instruction). The central and key part of the template contains the target instruction itself: in the case of N templating, similarly to previous work [71, 72], we surround the target instruction with a few NOPs to separate it from the setup phase, while in the case of R templating, we insert a random instruction before and after, making sure the control flow is not altered. Finally, at the end of the template code, we insert an instruction with an invalid opcode, to trigger a signal to the controller that the code execution is completed (see Fig. 3).

**Table 3:** RV32I base integer instructions for template generation

**Category**	**Instructions**
Arithmetic	ADD, ADDI, SUB, LUI, AUIPC
Logical	XOR, XORI, OR, ORI, AND, ANDI
Compare	SLT, SLTI, SLTU, SLTIU
Shifts	SLL, SLLI, SRL, SRLI, SRA, SRAI
Loads	LB, LH, LW, LBU, LHU
Stores	SB, SH, SW
Branches	BEQ, BNE, BLT, BGE, BLTU, BGEU
Jump & Link	JAL, JALR

### Instruction Classification Models

Most power side-channel disassemblers in previous work used traditional ML methods and common classification algorithms, e.g., quadratic discriminant analysis (QDA), *k*-nearest neighbors (k-NN), support vector machines (SVM), Gaussian Diffusion Model (GDM) [67, 69, 71–73]. However, the accuracy of these algorithm-driven ML classifiers dramatically depends on the preprocessing for dimensionality reduction and feature extraction. Without suitable preprocessing, the noise in the dataset can significantly affect the classification results. For these reasons, previous research relied on the high sampling rate of the oscilloscope to achieve reasonable accuracy. In this work, considering the limited sampling frequency of the on-chip sensors with respect to the soft-core CPU operating frequency, besides testing how well the ML methods proposed in previous work perform in this scenario, we explore leakage analysis using DL-based classifiers.

**Fig. 7:**
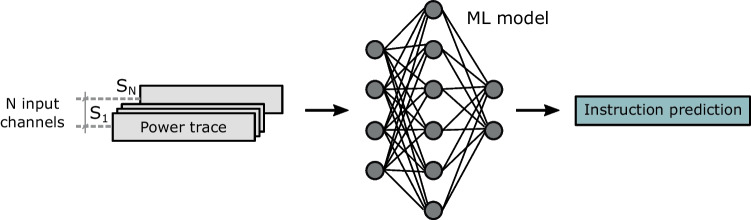
Classification process. The power trace of each sensor (*S*_1_ to *S*_*N*_) is used as one of N input channels. The input is then forwarded to the model, and the instruction prediction is collected for accuracy evaluation

First, we treat the side-channel instruction classification as a time-series classification problem, as different instructions have unique patterns spread across the time and amplitude domain. Since we use multiple sensors for classification, we represent the trace of each sensor as a separate input channel. Fig. 7 shows the classification process.

 A class of networks naturally suited to processing sequential data is recurrent neural networks (RNNs), specifically long short-term memory (LSTM) models [74]. They have an internal state that can represent context information, and they keep information about past inputs for an amount of time that is not fixed but depends on the weights and the input data. As LSTMs do not perform well when directly extracting features from raw data, they are commonly paired with more complex networks for feature extraction [75, 76], such as convolution neural networks (CNNs). In practice, feature extraction with CNNs can be applied before or after the LSTM model. Moreover, recent work showed that 1D-CNNs consisting of single-dimensional convolutional layers achieved good results in time-series classification [77]. Finally, CNNs structured as residual networks (ResNets) have shown to be very performant in time-series classification, achieving high accuracy across a range of datasets [77]. Therefore, we train and compare the following models: LSTM, a small 1D-CNN, a large 1D-CNN, and the combination of LSTM and 1D-CNN (LSTM followed by 1D-CNN and LSTM preceded by 1D-CNN), a multi-layer perceptron (MLP), and a time-series ResNet [77].

**Fig. 8:**
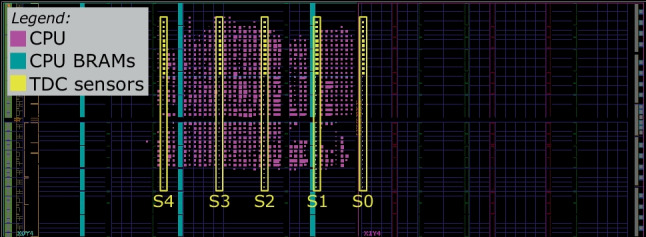
Sensor delay lines (in yellow) and CPU (in purple) in Exp-IN

## Evaluation on Sakura-X

In this section, we provide a detailed instruction-level leakage analysis on Sakura-X. The first step in experimental evaluation is deciding the hypothetical attacker and victim’s placement. Given the power delivery network imperfections and knowing that side-channel leakage picked up by the sensors varies with both the absolute and the relative positions of the victim and the attacker [66], we opt to assign the victim to an arbitrary FPGA region and vary the sensor placement.

**Fig. 9:**
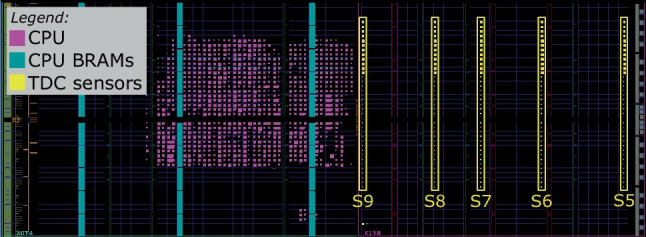
Sensor delay lines (in yellow) and CPU (in purple) in Exp-OUT1

**Fig. 10:**
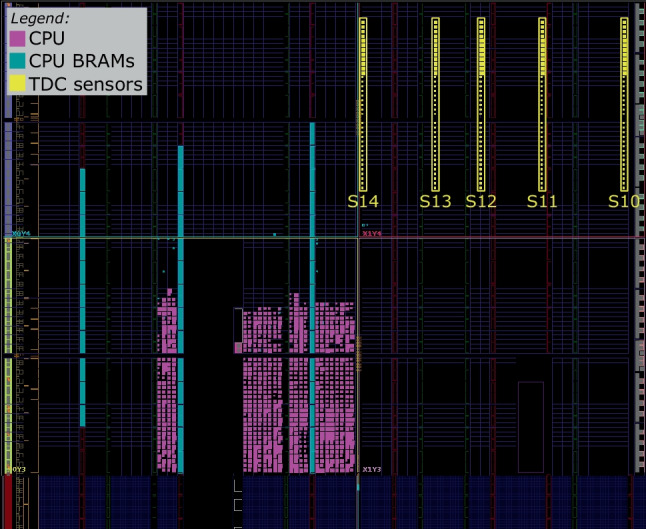
Sensor delay lines (in yellow) and CPU (in purple) in Exp-OUT2

Figures 8, 9, and 10 zoom in on the FPGA floorplan containing three different placements of the target CPU and the sensors. In the floorplan in Fig. 8, we place the sensors inside the region occupied by the target CPU, in the top-left clock region of the Kintex-7 FPGA (X0Y4). Even though this floorplan does not conform to the standard shared FPGA threat model—where the FPGA regions assigned to the tenants do not overlap—we use it as a worst-case leakage scenario for the evaluator (best-case scenario for the attacker). In the floorplan in Fig. 9, we place our five sensors to the right of the target—in the top-right clock region (X1Y4)—in the space between the CPU and the edge of the FPGA, simulating an attacker that spreads out the available sensors across their entire FPGA region. In the floorplan of Fig. 10, we move the target CPU one clock region down (X0Y3), further away from the sensors. In the remainder of this section, we will refer to the described floorplans as Exp-IN, EXP-OUT1, and Exp-OUT2, respectively.

Using the leakage evaluation setup and following the instruction classification method described in Sects. 4 and 5, we create 10,000 templates per instruction (for both N and R template types) and collect the corresponding power side-channel traces, creating four datasets: Exp-IN-N, Exp-OUT1-N, Exp-OUT1-R, and Exp-OUT2-N. We use the Exp-IN-N dataset to evaluate the worst-case leakage (i.e., with physical separation between the victim and the adversary violated and no noise of surrounding instructions). Exp-OUT1-N and Exp-OUT2-N are collected in addition to Exp-IN-N to evaluate the impact of CPU and sensor placement on the instruction-level leakage and model accuracy. Finally, we use Exp-OUT1-R to evaluate the most realistic scenario, where the templates contain the noise of the surrounding instructions. With these four datasets, we cover the three main goals of our instruction-level leakage evaluation: worst-case for the evaluator (Exp-IN-N), the impact of CPU and sensor placement on the accuracy (Exp-IN-N, Exp-OUT1-N, Exp-OUT2-N), and a realistic case for the attacker (Exp-OUT1-R).

We set the sensor trace length to $T = 60$ samples (i.e., 60 consecutive readings of the TDC output register), to guarantee that all the execution cycles of the instructions in Table 3 are captured. To ensure we are capturing the correct instruction execution, we align the start of all instructions to the same sample in the traces (fourth sample): we center the traces around the correct instruction using the recorded CPU opcode.

In our experimental evaluation, we first visually analyze the recorded power traces for multiple sensor placements. We show that instructions of different types show limited visual leakage patterns, while the instructions of the same type do not display any differences. To determine the limits of the instruction-level leakage, we train a range of DL models on the four datasets and show how the accuracy changes depending on the placement and template type. We also use ML techniques to further evaluate the inter- and intra-type instruction leakage and show that most of the classification confusion comes from two or three instructions with similar leakage. We show that preprocessing techniques and ML approaches used in previous work are outperformed by DL techniques. Finally, we evaluate the limits of the instruction-level leakage by investigating the impact of the number of sensors, averaging, and the dataset size on the accuracy.

### Visual Analysis of Sensor Traces

**Fig. 11:**
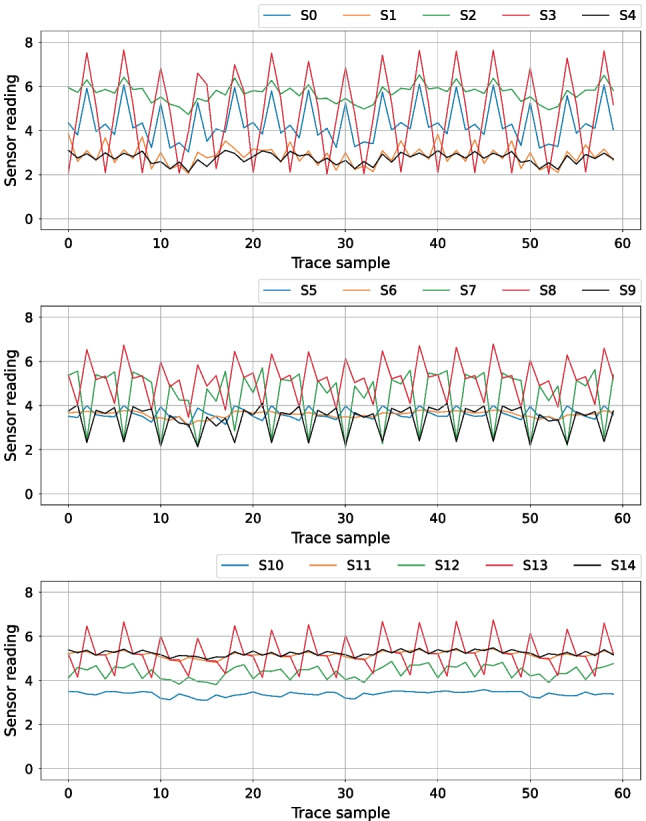
Average sensor traces for the arithmetic and logical instructions in Table 3

Before analyzing leakage using the DL-based classification methodology described in Sect. 5.2, we first visually analyze the recorded sensor traces. In our first experiment, we investigate how sensor placement impacts the waveforms and the leakage in the traces. Figure 11 shows the average trace of all templates of arithmetic and logical instructions across all 15 sensors (five in each of the three floorplans) for the Exp-IN-N, Exp-OUT1-N, and Exp-OUT2-N datasets. We can observe that the sensor placement significantly impacts the shape of the traces, including the peak-to-peak ratio: S10, the furthest from the CPU, has a peak-to-peak ratio of less than one, while S3 has a peak-to-peak ratio of almost six. For some sensors (e.g., S4, S8, S7, S12, and S13), we observe peaks every four samples, perfectly synchronized with the CPU clock. For some other sensors (e.g., S1, S2, and S4), we observe a different pattern: slight dips every 20 sensor samples (around samples 12, 32, and 52), corresponding to five CPU clock cycles, i.e., to the fetch of the next instruction. This experiment already shows the benefit of having multiple sensors for increasing the power side-channel leakage.

**Fig. 12:**
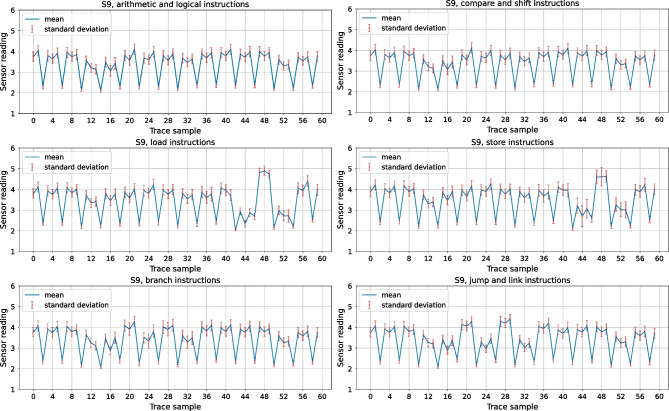
Average traces of sensor S9 for all instruction groups in Table 3

In our next experiment, we visually inspect the inter-type instruction leakage, i.e., how different instruction types impact the shape of the recorded side-channel traces. Figure 12 shows the average traces of sensor S9 (Exp-OUT1-N) for the six instruction groups in Table 3. We chose sensor S9, as the plots in Fig. 12 were most visually distinguishable for this particular sensor and it represents the worst-case scenario for an evaluator. The peak in sample 48 makes the load and store instructions clearly distinguishable from other groups. Branches and jumps also contain a distinguishable peak centered around sample 28, surrounded by dips on both sides. This experiment shows that, after significant averaging, some distinct visual traits can be attributed to specific instruction groups. However, not all instruction groups can be identified visually. For example, just like loads and stores, jumps and branches have very similar power consumption traces, and it is difficult to tell the exact instruction type from visual analysis alone.

**Fig. 13:**
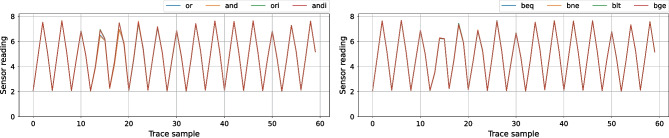
Average S3 traces for OR, AND, ORI, and ANDI (left) compared to S3 traces for BEQ, BNE, BLT, and BGE (right)

As the final visual experiment, we compare the side-channel traces of several instructions of the same instruction type. Figure 13 shows average sensor S3 traces for eight instructions. We chose S3 because it is in the heart of the soft-core CPU (Fig. 8), and it shows, when averaged, the biggest visual differences between instructions of the same type. On the left, we overlap the average traces for OR, AND, ORI, and ANDI. The differences, located between samples 10 and 20, are difficult to notice even with averaging across all templates, as all four instructions use the same datapath. On the right, we overlap the average traces of four branch instructions: BEQ, BNE, BLT, and BGE. We practically see no difference between these instructions and cannot distinguish them visually. Therefore, even though the visual classification of instructions is possible for some victims (e.g., sizeable ML-based accelerators [28]), soft-core CPUs require more advanced methods for instruction-level leakage analysis.

### Deep Learning-Based Instruction Leakage Evaluation

After showing that visual analysis is insufficient to identify CPU instructions executing on remote FPGAs, we deploy advanced DL techniques. We obtain our four datasets by collecting all the sensor traces for each instruction in Table 3, as described in Sect. 5.2. Each data point, corresponding to one instruction template, is represented as a matrix with five rows, where each row, i.e., the input channel, is the trace of one of the five sensors. Using the newly created dataset, we first train our deep learning models from Sect. 5.2 using 10-fold validation and compare the resulting accuracy. Then, we compare the results of our DL models with the classical ML methods previously proposed for side-channel disassembly attacks, and we evaluate if frequency-based preprocessing methods, shown promising in previous work [72], have any impact on the extracted leakage. Furthermore, we evaluate how the number of sensors used in the attack impacts the final accuracy. Finally, we evaluate how the amount of averaging or a smaller dataset size can impact the leakage, i.e., the best model accuracy.

To train our deep learning models, we set the number of epochs and the batch size to 100 and 64, respectively. We use the Adam optimizer with an initial learning rate of 0.0001 and the loss to monitor and adjust the learning rate. Table 4 summarizes the model details. To facilitate reproducibility, we choose deep learning models with standardized parameters and openly available implementations [77, 78].

**Fig. 14:**
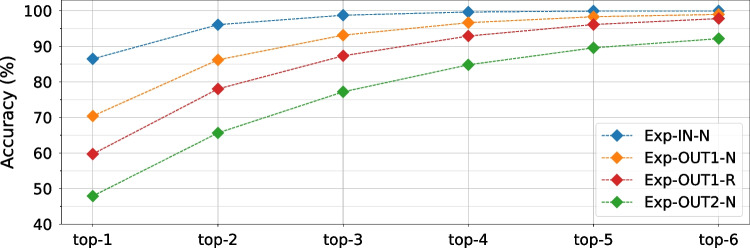
Top-K accuracy (K = 1, 2, 3, 4, 5, and 6) using ResNet, for all four datasets

**Table 4:** Architecture details of the deep learning models

Model	Architecture
MLP	Dense(X units, ReLU) $\vert$ X = (250, 350, 150, 50)
	Dropout(0.2)
	Dense(100, ReLU) + Dense(37, Softmax)
1D-CNN1	Conv1D(X filters, kernel size of Y) + MaxPool(2) $\vert$ (X,Y) = ((64,10), (64, 4))
	Dropout(0.2)
	Dense(100 units, ReLU) + Dense(37, Softmax)
1D-CNN2	Conv1D(X filters, kernel size of Y) + MaxPool(2) $\vert$ (X,Y) = ((32,12), (45, 10), (64,8), (128,4))
	Dropout(0.2)
	Dense(100 units, ReLU) + Dense(37, Softmax)
LSTM	LSTM(100 units)
	Dropout(0.2)
	Dense(100 units, ReLU) + Dense(37, Softmax)
1D-CNN & LSTM	Conv1D(64 filters, kernel size of 4, ReLU)
	Conv1D(64 filters, kernel size of 4, leakyReLU=0.3)
	Dropout(0.2)
	MaxPool(2)
	LSTM(100 units)
	Dense(100 units, leakyReLU=0.3) + Dense(37, Softmax)
LSTM & 1D-CNN	LSTM(100 units)
	Conv1D(64 filters, kernel size of 2, leakyReLU=0.3) + MaxPool(2)
	Dropout(0.2)
	Dense(100 units, leakyReLU=0.3) + Dense(37, Softmax)
ResNet	Standard time-series Resnet: 3 blocks with 3$\times$Conv1D layers and residual connections [77]

Table 5 lists the average test accuracy obtained with the four datasets, with the highest accuracy in bold. We can observe that overall, ResNet and 1D-CNN2, the two most complex DL models, achieve the highest accuracy for all datasets. Results in Table 5 also show that models without convolutional layers do not manage to extract leakage well and result in low classification accuracy. Moreover, the accuracy drops as the sensors are placed further away from the target CPU. For example, for the best model (ResNet), Exp-OUT1-N has a 16.07% lower accuracy than Exp-IN-N, and Exp-OUT2-N has a 22.5% lower accuracy than Exp-OUT1-N. Therefore, an evaluator testing local CPU leakage with sensors placed inside the CPU will achieve an overestimation of the leakage—if leakage does not exist in a scenario such as Exp-IN-N, an evaluator can, with a high probability, guarantee that a potential attacker will not be able to exploit the leakage. Finally, Table 5 also shows the contrast in accuracy resulting from differences between isolated instructions (Exp-OUT1-N) and instructions with random instructions surrounding them (which is the case in a code sequence execution). We can observe that the drop in SNR caused by additional instructions results in a 10.69% accuracy drop for the ResNet model.

**Table 5:** Instruction classification accuracies (in %) for the deep learning methods. The highest accuracies, in bold, are obtained using the 1D-CNN2 and ResNet models

**Dataset**	**Average Accuracy (%)**						
	**MLP**	**1D-CNN1**	**1D-CNN2**	**LSTM**	**1D-CNN & LSTM**	**LSTM & 1D-CNN**	**ResNet**
Exp-IN-N	79.24	82.38	84.91	77.21	81.53	84.04	**86**.**46**
Exp-OUT1-N	61.60	65.15	69.58	61.63	65.07	68.45	**70**.**39**
Exp-OUT1-R	52.89	56.79	59.10	50.58	55.59	58.44	**59**.**71**
Exp-OUT2-N	43.29	46.11	**48**.**03**	42.10	44.70	46.84	47.89

For the best model, i.e., ResNet, we explored different hyperparameters with the goal of increasing the accuracy of Exp-OUT1-R, a realistic dataset in terms of an attack. Increasing the initial learning rate and the number of epochs did not result in higher accuracy. An increase of the batch size to 128 or a reduction to 32 did not significantly change the classification accuracy. Increasing the number of ResNet blocks, increasing the number of layers per block, or adding an LSTM layer at the input did not significantly change the ResNet classification accuracy either.

To analyze further the instruction-level leakages and understand why the classification accuracy for Exp-OUT1-R does not reach a number significantly higher than 60%, we evaluate the top-K accuracy of our best model for all four datasets. Unlike the regular model accuracy, i.e., top-1 accuracy, the top-K accuracy labels a prediction as correct if the real class is among the top K predicted classes (ranked by predicted scores). If the top-K accuracy is high while the top-1 accuracy is low, this signifies that groups of classes are often confused. Figure 14 shows the top-K accuracy for ResNet (using 10-fold validation), for all four datasets, and K ranging from one to six. We can observe that, for all datasets, the trend is the same, and the accuracy significantly increases with K. The most significant accuracy increase is observed between top-1 and top-2 accuracy: 15% on average. The difference reduces for every subsequent K increase while the accuracy converges to almost 100% for all datasets except Exp-OUT2-N. This trend shows that the main difficulty for the classification is distinguishing between two or three similar instructions. For Exp-OUT2-N, the sensors are far away from the soft-core CPU and record a limited leakage compared to the other two placements, which is also noticeable in the weaker visual trace properties in Fig. 11.

**Fig. 15:**
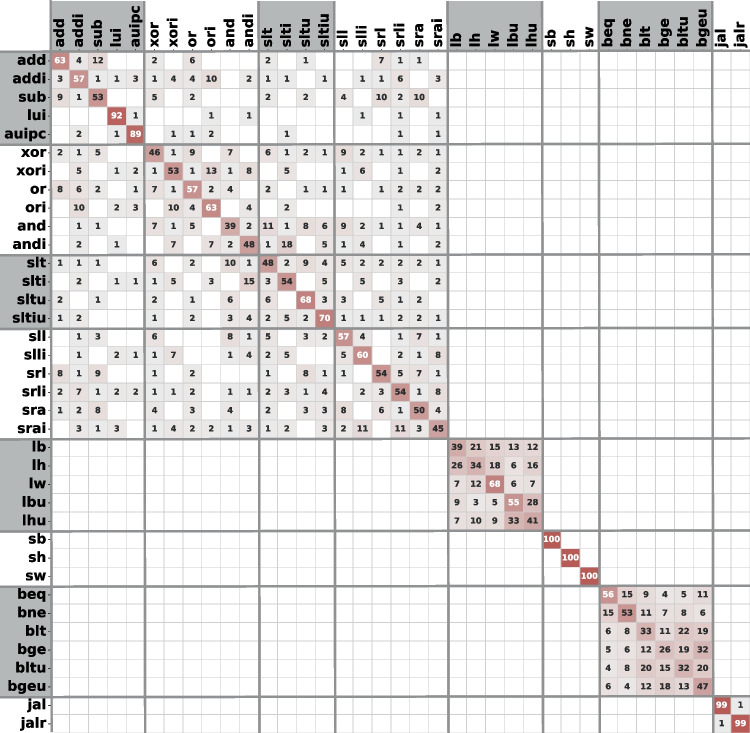
Normalized confusion matrix (in %, rounded) of the ResNet model (100 epochs), for Exp-OUT1-R

To evaluate which instructions have similar leakages and lower the top-1 accuracy, we look into how well ResNet distinguishes between the instructions in Table 3. The corresponding normalized confusion matrix is shown in Fig. 15. We see that instructions of a similar type are more challenging to tell apart; for example, different branch instructions. Other examples include arithmetic, shift, and logical operations. The confusion is not surprising, as many instructions share the CPU datapath and, consequently, tend to have very similar power consumption patterns, which makes the classification task harder. However, it is worth noting that the classification is highly successful for instructions of different types, allowing a potential attacker to easily distinguish between the control and data flow of the executed code sequence. This is also confirmed by the 100% top-6 accuracy in Fig. 14, and the visual analysis presented in Sect. 6.1.

As our final instruction leakage evaluation experiment, we implement a hierarchical approach to instruction classification. We first perform inter-type classification—training the ResNet model to classify between different instruction types—and then we perform intra-type classification by training a ResNet per each instruction type. Table 6 shows the results of inter- and intra-type classification using 10-fold validation on Exp-OUT1-R. We can observe that the inter-type accuracy is significantly (>20%) higher than the ResNet accuracy on the entire dataset, as the model does not need to classify between similar instructions of the same type. Furthermore, we can see that the intra-type classification accuracy heavily correlates with the confusion shown in Fig. 15: instruction types with high intra-type confusion in Fig. 15 such as loads and branches, have very low intra-type classification accuracy in Table 6.

**Table 6:** Classification accuracy of the ResNet model trained for hierarchical classification on the Exp-OUT1-R dataset

**Average Hierarchical Classification Accuracy (%)**								
**Inter-Type Classification**	**Intra-Type Classification**							
83.60	Arithmetic	Logic	Compare	Shift	Load	Store	Branch	Jump
	86.77	72.03	81.35	70.31	49.14	100	39.62	99.20

**Fig. 16:**
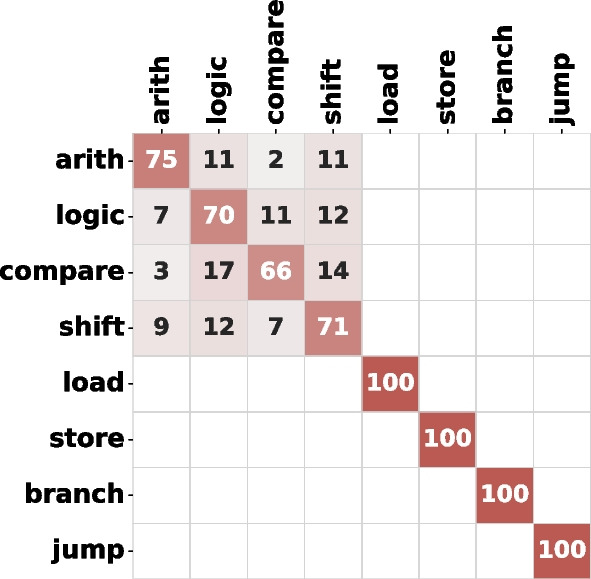
Confusion matrix in case of instruction type classification

Finally, Fig. 16 shows the confusion matrix of the inter-type classification. Similar to Fig. 15, there is no confusion between loads, stores, branches, and jumps. The confusion is limited to arithmetic, logic, shift, and compare instructions, which are ALU instructions and share most of the processor datapath.

### Impact of Preprocessing On Instruction Leakage

Previous work showed the importance of preprocessing for increasing the classification accuracy when identifying instructions [72, 73, 79, 80]. Furthermore, frequency-domain analysis, particularly the continuous wavelet transform (CWT), was shown to be well-suited for side-channel disassembly [72]. Therefore, we evaluate our deep learning models with CWT preprocessing to determine if CWT is beneficial for extracting instruction-level leakage in our setting.

From a time-series vector of *M* sampling points, CWT creates a matrix of $M \times D$ entries, where the *D* dimensions describe how *D* frequency components of the time series change over time. Including the original time-series vector in the CWT matrix results in a matrix of $M \times (D+1)$ entries. Knowing that every entry in our original dataset contains five sensor traces, each having *T* samples, we create the following two additional datasets using CWT with a scale parameter of *D*. We perform CWT on each sensor trace individually, resulting in five $T\times (D+1)$ matrices for each data point. For the first dataset, *CWT-H*, we concatenate these matrices horizontally in a $5T\times (D+1)$ feature matrix. For the second dataset, *CWT-V*, the matrices are concatenated vertically, resulting in a $T\times 5(D+1)$ feature matrix.

Table 7 shows the average 10-fold validation accuracy drop (compared to the baseline datasets in Table 5) using DL models with CWT. The scale parameter *D* for CWT is set to 49 [72]. As can be seen, most DL models do not benefit from the increase of the input space size. For models with high accuracy in Table 5 such as ResNet and 1D-CNN2, the overall accuracy drops on average by approximately 2–3%, with a maximum drop being 16.05% for 1D-CNN & LSTM with CWT-H. For models with originally low accuracy in Table 5 such as MLP and LSTM, preprocessing slightly increases the accuracy: for approximately 2–3%. We can therefore conclude that with well-fitted models, a DL approach does not require computationally heavy CWT preprocessing, as the models are complex enough to capture the correlation between the traces and the instructions.

**Table 7:** The increase in the instruction classification accuracies for the deep learning methods when using dataset preprocessing with CWT compared to no preprocessing

	**Average Accuracy Increase (%)**								
**Model**	Exp-IN-N		Exp-OUT1-N		Exp-OUT1-R		Exp-OUT2-N		
	CWT-H	CWT-V	CWT-H	CWT-V	CWT-H	CWT-V	CWT-H	CWT-V	Average
**MLP**	3.00	3.01	3.87	4.18	1.74	1.63	2.32	2.36	2.76
**1D-CNN1**	–0.70	–0.27	–1.98	–0.05	–3.67	–1.93	–2.59	–1.04	–1.53
**1D-CNN2**	–2.40	–1.92	–4.59	–2.72	–4.95	–2.73	–4.39	–2.07	–3.22
**LSTM**	0.05	3.81	0.54	3.28	0.11	3.42	1.15	2.74	1.88
**1D-CNN & LSTM**	–13.35	–3.25	–16.05	–3.41	–10.23	–2.23	–5.23	–0.95	–6.84
**LSTM & 1D-CNN**	–0.56	–0.69	–2.56	–1.57	–3.43	–2.24	–2.78	–1.62	–1.93
**ResNet**	–3.66	–2.04	–5.19	–2.37	–4.77	–2.25	–3.04	–2.01	–3.17

### Comparison with Classical ML Approaches

Physical power side-channel disassembly attacks relied on high-frequency oscilloscopes and classical ML techniques to achieve high profiling accuracy. However, in our work, we use TDC sensors sampling at 320 MHz, having a significantly lower sampling frequency than oscilloscopes. To evaluate how disassembly techniques used in previous work translate on TDC sensor traces, we obtain the instruction classification accuracy using common ML models (GDM, QDA, k-NN, and SVM) and preprocessing techniques—principal component analysis (PCA) and linear discriminant analysis (LDA)—used in previous work on side-channel disassembly, discussed in Sect. 11.2. Table 8 lists the average 10-fold validation accuracy of classical ML approaches. We obtain the highest accuracy using SVM with PCA and QDA with LDA: 68.74% for Exp-IN-N, 52.45% for Exp-OUT1-N, 47.06% for Exp-OUT1-R, and 37.65% for Exp-OUT2-N, which is 10–20% lower than the accuracy of our best-performing DL-based classifier. Even the deep learning models with lower accuracy (LSTM, 1D-CNN & LSTM) are comparable with the best results in Table 8. We can, therefore, conclude that advanced techniques, such as deep learning, are required in the shared-FPGA attack scenario, as it involves low resolution and a reduced sampling rate of the voltage sensors coupled with a high victim CPU frequency.

**Table 8:** Instruction classification accuracies for the classical machine-learning methods. The highest accuracies, in bold, are obtained when combining SVM with PCA and QDA with LDA

**Method**	**Average Accuracy (%)**							
	PCA				LDA			
	GDM	QDA	k-NN	SVM	GDM	QDA	k-NN	SVM
Exp-IN-N	56.55	67.15	39.64	**68**.**74**	59.78	**67**.**17**	50.28	65.33
Exp-OUT1-N	45.36	48.56	26.87	**51**.**83**	48.24	**52**.**45**	36.15	50.44
Exp-OUT1-R	39.66	41.80	22.02	**47**.**06**	43.31	**46**.**47**	30.59	45.96
Exp-OUT2-N	33.74	34.23	26.63	**37**.**65**	34.82	**37**.**19**	29.02	37.11

### Impact of the Number of Sensors on Instruction Leakage

To investigate the role of the number of sensors in the attack, we analyze the impact of incrementally including additional sensors in the dataset on the classification accuracy. The analysis is performed for all four datasets and on sensor data collected in the setup where all sensors are simultaneously present. Since the sensors record power traces of the same events simultaneously, they are subject to the same experimental conditions (e.g., environmental noise or temperature), facilitating a fair comparison. Furthermore, as the power-intensive measurement logic (memory and controllers) is placed far from the sensors, only the last few elements in their 16-bit delay lines cause differences in sensors’ switching activity, which is thus negligible compared to the switching activity of the CPU.

In this experiment, we choose our best-performing model: ResNet. We start by training separate models, one for each sensor, and evaluate the instruction classification accuracy. Table 9 summarizes the results. We can observe that the closest sensor does not necessarily have the highest classification accuracy—as sensor S7 has higher accuracy than S5—which is in line with conclusions from previous work [66]. However, we can observe that the further the sensors are from the soft-core CPU, the smaller the accuracy difference between the best and the worst sensors: for Exp-IN-N and Exp-OUT1-N the range is 10–20%, while the sensors in Exp-OUT2-N have a maximum difference of 2–3%. This signifies that across multiple sensors, the overall placement does have an impact on the accuracy, also confirmed by results in Table 5.

**Table 9:** Average instruction classification accuracies (in %) of ResNet, when trained on the traces of a single sensor only. In bold, the highest accuracies for each of the four datasets

**Average Accuracy (%)**														
Exp-IN					Exp-OUT1-: N (top), R (bottom)					Exp-OUT2-N				
**S0**	**S1**	**S2**	**S3**	**S4**	**S5**	**S6**	**S7**	**S8**	**S9**	**S10**	**S11**	**S12**	**S13**	**S14**
48.76	60.22	64.59	**71**.**52**	53.14	39.32	40.52	**55**.**50**	45.00	44.34	39.78	41.24	41.79	**42**.**38**	39.69
					31.17	31.54	**45**.**86**	36.92	38.16					

**Fig. 17:**
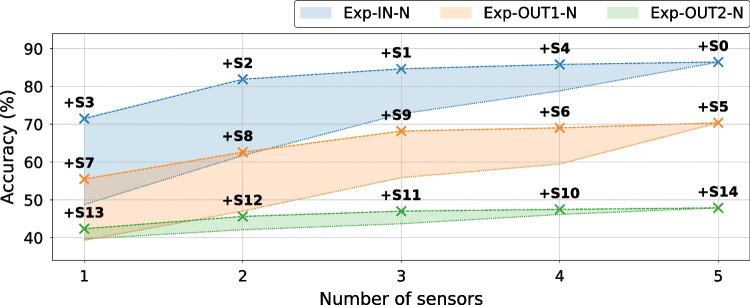
Average instruction classification accuracy in the function of the number of sensors contributing to the dataset, for all datasets, with the ResNet model. Upper, dashed lines correspond to including the next best sensor in the dataset. Lower, dotted lines correspond to including the next worst sensor in the dataset

Using data from Table 9, we sort the sensors by the obtained accuracy, once in increasing order (from “worst” to “best”) and once in decreasing order (from “best” to “worst”). Figure 17 illustrates the accuracy increase in function of the number and the choice of sensors in the dataset used for training. The dashed (respectively, dotted) lines show the accuracy increase when the next best (respectively, next worst) candidate sensor is added to the dataset. For example, the highest accuracy achieved with a single sensor (71.52% in Fig. 17) corresponds to sensor S3 and Exp-IN-N (Table 9), while the accuracy obtained after adding the next best candidate (81.91% in Fig. 17) corresponds to sensors S3 and S2 used together. The accuracy increase is more pronounced on the dotted lines, as every new sensor added to the dataset has better individual accuracy than the ones already in the dataset. Comparing the trend of the shaded regions, we see that the sensors in Exp-OUT1-N and Exp-OUT2-N floorplans, being further away from the CPU, pick up less information leakage. However, the distance between the best and worst-case region borders reduces significantly when four or five sensors are used, showing the importance of using multiple sensors for better leakage extraction. Finally, we can observe that the shaded region for Exp-OUT2-N is significantly narrower than for the other two datasets in Fig. 17: an effect that arises because the range between the best and the worst sensor for Exp-OUT2-N is significantly smaller than for Exp-IN-N and Exp-OUT1-N.

### Impact of Averaging on Instruction Leakage

**Fig. 18:**
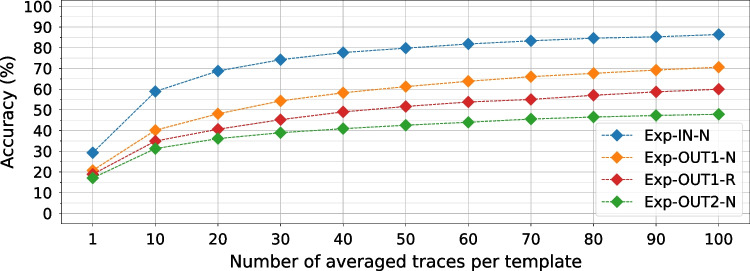
Accuracy in the function of the number of averaged traces per template. The dataset size is 10,000 templates per instruction, while the model used for training is ResNet

To evaluate the impact of averaging on leakage and the ability of DL models to extract it, we use the best model, ResNet, and train it on the four datasets while changing the number of traces averaged for each template. Figure 18 shows the results. We can observe that with only a single trace (no averaging), all four datasets have very low accuracy: approximately 30% for Exp-IN-N and 20% for the other three datasets. Note that, in the beginning, as we increase the number of averaged traces, the accuracy increases significantly for all four datasets, showing the benefit of averaging in eliminating noise. This experiment also shows that increasing the averaging does not indefinitely increase the SNR, as the curves in Fig. 18 are logarithmic and flatten off after using roughly 80 averaged traces per template.

From a leakage evaluator point of view, we can see the usefulness of averaging for identifying and analyzing instruction-level leakages. As trace acquisition is a time-consuming process, we see that finding a good number of traces for averaging can help reduce the evaluation time. From an attacker’s point of view, we can observe that recording only one trace of the victim execution might not be sufficient for extracting secret information: the attacker might have to resort to recording multiple victim executions or folding loops to accommodate averaging for a better SNR.

### Impact of the Dataset Size on Instruction Leakage

**Fig. 19:**
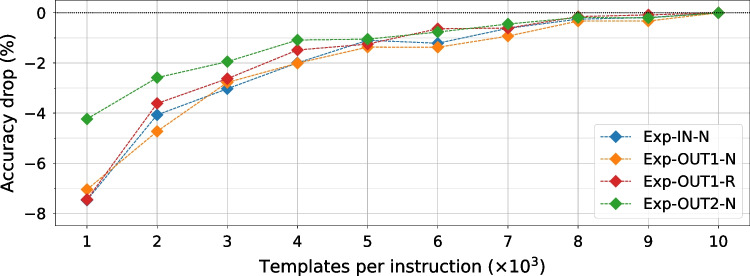
Accuracy drop in the function of the dataset size (number of templates) used for training and testing compared to the full dataset with 10,000 templates. Results are shown on the ResNet model for all four datasets

As our final experiment on the Sakura-X board, we evaluate the impact of the number of templates per instruction, i.e., the dataset size, on the accuracy of the ResNet model. For five different input seeds, we randomly select a subset of the templates for each instruction and train the ResNet model. Figure 19 shows the accuracy drop compared to the full dataset for a range of template sizes, averaged across all five seeds. We can observe that when using only 10% of the dataset size, i.e., 1000 templates per instruction, the accuracy of Exp-IN-N drops only 4%, while it drops approximately 7% for the Exp-OUT datasets. This indicates that increasing the accuracy is a very difficult problem, as the initial accuracy comes from the inter-type classification, and the intra-type confusion cannot be significantly improved even by increasing the dataset size by 10$\times$. Figure 19 also shows that after some point, as with averaging, the accuracy does not significantly increase with a bigger dataset size. We can observe that the curves flatten for all four datasets and that using more than 8k templates does not significantly affect the accuracy.

## Evaluation on Alveo U200

**Fig. 20:**
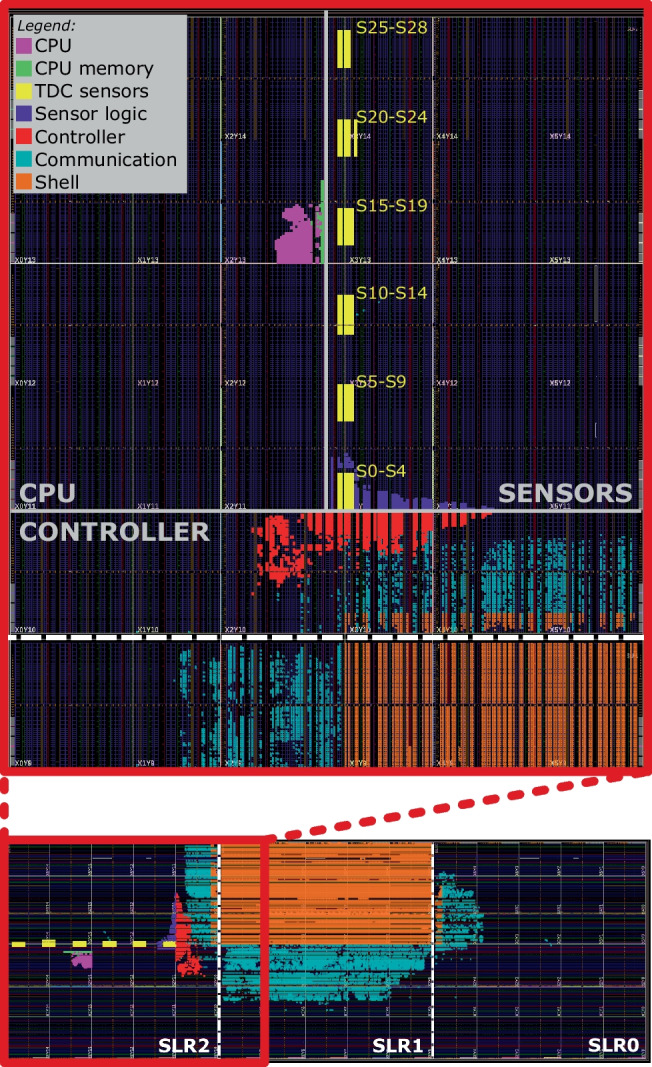
Floorplan on the Alveo U200 board. Sensor delay lines (in yellow) and CPU (in purple)

This section provides an instruction-level leakage analysis on the Alveo U200 board containing a cloud-scale AMD Virtex Ultrascale+ FPGA. Figure 20 shows the floorplan. At the bottom, we can see the entire FPGA rotated by 90°, with three SLRs, and the shell occupying half of the middle SLR. Figure 20 also shows the enlarged view of SLR2. Similar to the Exp-OUT1 placement in Fig. 9, we physically separate the soft-core CPU (PicoRV32 [11]), the sensor region, and the controller. As Alveo U200 contains a much larger FPGA than Sakura-X, we instantiate 29 sensors, as described in Sect. 4. However, unlike the spread-out placements in Exp-OUT1 and Exp-OUT2, we place the 29 sensors along the border of the sensor region, clustered in 6 equidistant groups of five sensors (except the last group with four sensors), simulating an attacker placing all the sensors as close to the victim as possible.

Using the leakage evaluation setup and following the instruction classification method described in Sects. 4 and 5, we create 10,000 templates of N type and 20,000 templates of R type. We collect the corresponding power side-channel traces, creating three datasets: Exp-10k-N, Exp-10k-R, and Exp-20k-R. Since the sensor and the soft-core CPU both work at the same clock frequency, the sensor traces do not need as many samples as for the Sakura-X board: we set the sensor trace length to $T = 16$ samples which guarantees that the longest instruction execution is completely captured. Like in the Sakura-X experiments, we align the start of all instructions to the same sample in the traces using the recorded CPU opcode.

### Instruction-Level Leakage on Cloud-Scale FPGAs

In the first experiment on Alveo U200, we evaluate the instruction-level leakage by training the ResNet model on all three datasets. Table 10 shows the averaged results for 10-fold validation. We can observe that on a significantly larger FPGA and a CPU running at the same high clock frequency as the sensor, the accuracy for all three datasets is approximately 40%. The Exp-OUT-20k-R dataset has a 20% lower accuracy than the Exp-OUT1-R dataset on Sakura-X, while the Exp-OUT-10k-N dataset has an approximately 30% lower accuracy than the Exp-OUT1-N dataset. Table 10 also shows that the difference between N and R datasets is much smaller on Alveo U200 ($\approx$5%) compared to Sakura-X ($\approx$10%). As the target used on Alveo is a multicycle CPU (PicoRV32 [11]) and the target used on Sakura-X is a pipelined CPU (RISC-Y [32]), the impact of surrounding instructions is smaller on a multicycle CPU. Finally, Table 10 confirms the findings shown in Sect. 6.7, as the accuracy difference when using 10,000 templates (Exp-OUT-10k-R) and 20,000 templates (Exp-OUT-20k-R) is less than 2%: after a certain threshold, increasing the templates does not significantly impact the model’s ability to extract leakage.

**Table 10:** Instruction classification accuracies on Alveo U200 (in %) for the ResNet model

Model	**Average Accuracy (%)**		
	**Exp-OUT-10k-N**	**Exp-OUT-10k-R**	**Exp-OUT-20k-R**
ResNet	42.59	37.60	39.05

### Code Sequence Classification

As our final experiment, we evaluate the leakage of code sequences instead of single instructions. To do this, we train a classifier to predict which sequence was executed from a set of known code sequences. We create eight code sequences, each comprised of multiple instructions of the same type, separated by instructions of another type. Each code sequence has a primary type from Table 3: load, store, branch, arithmetic, logic, compare, and shift. For example, the load code sequence consists of load instructions separated by shift instructions, and the store code sequence contains store instructions separated by logic instructions. The number of instructions is tailored so that all eight sequences have the same execution length of 40 clock cycles. This structure makes them representative of short code sequences dominated by same-type instructions, commonly found in open-source code.

The code sequence templates have the same structure as instruction templates of type N in Algorithm 1, where instead of a single instruction, the target is a fixed set of instructions for the given sequence. For each code sequence, we create 10,000 templates, each representing an execution of the code sequence on random data. For this dataset, called Exp-10k-S, the traces for each template are recorded as an average across 1,000 executions of the same code sequence and input data.

Table 11 shows the average accuracy across five different seeds for all the DL methods in Sect. 5. We can observe that unlike instruction-level leakages, which contain the randomness of the operands and data as noise, code sequences emit significantly higher leakage, as almost all the models achieve high accuracy: 1D-CNN1, 1D-CNN2, LSTM & 1D-CNN, and ResNet achieve the accuracy of 100%. The only model with a noticeably low accuracy is LSTM since it fails to converge for four out of five seeds, while for the remaining seed, it achieves 80% accuracy.

**Table 11:** Code sequence classification accuracies (in %) for the deep learning methods. The highest accuracies, in bold, are obtained using the 1D-CNN1, 1D-CNN2, LSTM & 1D-CNN, and ResNet models

**Average Accuracy (%)**						
**MLP**	**1D-CNN1**	**1D-CNN2**	**LSTM**	**1D-CNN & LSTM**	**LSTM & 1D-CNN**	**ResNet**
99.45	**100**	**100**	26.82	82.63	**100**	**100**

From an evaluator’s point of view, this experiment shows that it is important to evaluate not only instruction-level leakages but also the deployed code in its entirety. Moreover, since short code sequences dominated by same-type instructions are common in open-source code, our results demonstrate that known, i.e., open-source code sequences can be profiled and more easily distinguished than single instructions. For example, in an AES algorithm, the attacker can use a load-intensive piece of code for profiling and easily differentiate it from a branch-intensive code sequence in a control-flow algorithm. To avoid potential exploits, users should deploy countermeasures or use proprietary (unknown) code. From an attacker’s point of view, these results show that attacking code sequences instead of individual instructions requires less attack effort for a potentially higher benefit.

## Discussion

In Sects. 6 and 7, we have seen the evaluation of instruction-level leakages on two FPGA boards. Unlike large ML accelerators—which require recording long execution traces and have significant architecture- and data-dependent power variations—the instructions of soft-core CPUs have very short execution traces: in the range of tens of microseconds. Consequently, our results show that soft-core CPUs do not have visible leakage in power traces that SPA can exploit; unless extensive averaging of a million traces is performed. Through visual analysis of averaged traces, we have observed that instructions of different types are more likely to have different leakages. In contrast, instructions of the same type have almost no differences despite averaging. These results are also confirmed by DL models, as both the confusion matrix and hierarchical classification indicate that the classification confusion is concentrated within instruction groups, not between them. Furthermore, a significantly higher top-K accuracy also demonstrates that the confusion between only a few instructions prevents the models from achieving 100% accuracy.

Our analysis demonstrates that for the evaluator’s worst-case scenario, i.e., an attacker breaching the physical separation barrier, the highest achieved accuracy is 86.46% using the time-series ResNet model [77]. We show that classical ML approaches used in previous side-channel disassembly work do not transfer well to the shared FPGA scenario. As no high-end oscilloscope equipment is available, using ML and preprocessing approaches from previous work on sensor traces results in a 10–20% lower accuracy than DL approaches.

Our results indicate that the templating impacts SNR and the model accuracy, where Exp-OUT1-N has an approximately 10% higher accuracy than the Exp-OUT1-R dataset. These results suggest that the evaluator should use N templating for the worst-case estimate, while for a more realistic estimate, they should use R templating.

Throughout our experimental evaluation, we show that placement does matter: the overall distance between the sensors and the CPU impacts the SNR and, thus, the accuracy. We demonstrate that increasing the distance between the CPU and sensors (Exp-IN, Exp-OUT1, Exp-OUT2) incurs an approximately 15% accuracy drop. For one sensor placement, our results, like previous work [66], also indicate that sensors closer to the CPU do not necessarily have the highest accuracy, possibly due to the imperfections of the PDN implementation and different sensor calibrations. Additionally, we show the benefit of using multiple sensors: the accuracy increases significantly ($\approx$15% for Exp-IN-N) when using five sensors instead of only one.

We analyze the impact of averaging on the ResNet model accuracy. We demonstrate that no averaging, i.e., only one trace recording per template, results in very low accuracy due to noise. From the point of an evaluator, averaging increases SNR, which reflects in our results, showing that averaging 80 traces can significantly increase the accuracy; however, averaging more traces brings limited to no further benefit. This means that an evaluator can use averaging for a worst-case leakage analysis, to identify weak points of their soft-core CPU design, while also knowing an attacker would need to deploy additional techniques, such as loop folding, to be able to average a single code execution trace. We find that increasing the dataset size does not significantly impact the accuracy: having 10$\times$ more templates increases the accuracy at most 8% (for Exp-OUT datasets), showing again that inter-type instruction classification is a relatively easy classification problem, achieving a specific accuracy with only 1,000 templates per instruction. In contrast, distinguishing between instructions of the same type is a hard classification problem, where even 10$\times$ more templates are insufficient for increasing the accuracy significantly.

Our results show that cloud-scale FPGAs exhibit less leakage due to their size and PDN structure. Consequently, the accuracy on both N and R template types is approximately 40%, significantly lower than on Sakura-X. However, unlike instructions, we demonstrate that short code sequences have significant leakage and that DL models can predict them with an accuracy of 100%.

Finally, our experimental analysis shows that to ensure no exploitable leakage, the evaluator should always test the worst-case scenario: multiple sensors with no physical separation, using N-type templates on a smaller FPGA with higher SNR, and averaging. In this case, the evaluator will either ensure there is no leakage or, if there is, they will be able to analyze it more efficiently and design appropriate mitigations.

## Countermeasures

Countermeasures against power side-channel analysis have been extensively studied, and they fall into two main categories: *hiding* and *masking* [70]. Hiding aims to reduce the SNR of the signal recorded by the attacker. Therefore, protections can either focus on reducing the leakage signal, e.g., by equalizing the data-dependent power consumption [81], or increasing the side channel noise. Because most attacks depend on aligned traces, hiding can also be done in the time dimension, by adding random delays or clock jitters during the hardware execution. Masking, on the other hand, requires processing algorithmically-randomized data, while maintaining the correctness of the circuit operation [82]. Both hiding and masking, however, suffer from considerable area overhead and vulnerability to higher-order attacks [70].

Mitigations for power side-channel disassembly attacks involve restructuring the code or redesigning the hardware to reduce leakage [83]. De Mulder et al. integrated defenses into the microarchitecture of a soft-core RISC-V processor and tested them on a Zynq FPGA [84]. They enhanced the side-channel security by protecting memory accesses and introducing masking in the CPU. Another example of a side-channel protected microprocessor is PARAM, developed and tested on a Sakura-X FPGA [85]. After analyzing the RTL and leakage of an open-source RISC-V processor, the authors used obfuscation to reduce datapath leakage and to conceal the addresses sent to the cache. Alternative (or complementary to) hardware changes are software defenses: random code injection, code obfuscation [86], or shuffling the instruction execution [87] are most used to protect proprietary code against side-channel disassembly attacks.

On shared FPGAs, protections against side-channel analysis commonly deploy different hiding techniques, better tenant isolation, or methods that prevent the deployment of sensor circuits. As hiding techniques, the works of Le Masle et al. [88] and Krautter et al. [89] are most relevant. Le Masle et al. designed a network of on-chip RO-based sensors to control power wasters and maintain a constant power consumption, thus reducing the SNR [88]. They used a proportional-integral-derivative (PID) controller as the control circuit, while power wasters were implemented using long routing wires (equivalent to high capacitive load). Similarly, Krautter et al. designed an active *fence* composed of ring oscillators placed between two neighboring FPGA tenants [89]. The actuator controlling the fence in a closed-loop control system was a TDC sensor, and the fence area overhead was 100% compared to the unprotected design.

Additionally, Güneysu and Moradi proposed a set of countermeasures on FPGAs [90]. Using BRAM write collisions, short circuits, and shift register LUTs, they implemented Gaussian noise to reduce the SNR. Sasdrich et al. improve the resistance against FPGA side-channel attacks by dynamically changing the hardware implementation of a PRESENT cipher at runtime using the FPGA partial reconfiguration [91]. All these countermeasures are independent of the design under protection and can hence be used to increase the side-channel security of soft-core CPUs in a shared FPGA scenario.

The final way of preventing remote power side-channel attacks on shared FPGAs is by detecting and forbidding sensor-like structures in the RTL designs: Krautter et al. [92] and La et al. [93] developed bitstream scanners, which search for *signatures* of potentially malicious circuits. Deploying them on the cloud could prevent remote attackers from recording power traces and thus achieve power side-channel security of soft-core CPUs. However, bitstream scanners are not 100% effective in preventing malicious designs, as researchers have found ways to implement stealthy voltage sensors using benign circuits [65].

## Limitations and Future Work

In this work, we experiment with two lightweight soft-core CPUs commonly used for embedded bare-metal applications, which support straightforward integration with FPGA logic. Within the spectrum of embedded CPU microarchitectures, we have focused on two prevalent varieties commonly used in previous work on power disassembly attacks: a multicycle and a pipelined CPU. Therefore, the results and conclusions in Sects. 6 and 7 should generalize to a good number of embedded CPU implementations and ISAs.

Our results show that inter-type instruction leakage is the strongest, while it is harder to distinguish instructions of the same type. This result implies that instructions using different hardware and datapath in the CPU, typically instructions of different types, exhibit varied leakage, thus rendering them more distinguishable by machine learning models. For instance, logic and arithmetic instructions solely utilize the ALU, while loads additionally fetch data from memory. Branches use the ALU and modify the program counter, and jumps merely alter the program counter. Conversely, instructions sharing most of the datapath exhibit similar leakage, making them difficult to distinguish. For example, for instructions where only the ALU opcode differs—such as logic instructions—the CPU controller executes the same steps, with only a different ALU operation. Regardless of the microarchitecture and ISA, these observations hold. Some microarchitectures might be single-cycle, some multicycle, and some pipelined. However, the overall impact of the microarchitecture is on the SNR, resulting in the leakage (and classification accuracy) being stronger or weaker, but not impacting our conclusions. For example, pipelined architectures might have a more significant difference between N and R datasets, while multicycle architectures might have a comparably smaller difference (as is the case of RISC-Y and PicoRV32).

Our insights on DL superseding classical ML approaches in cases with low SNR are also general and should not depend on the CPU microarchitecture. Similarly, our conclusions regarding placement, averaging, and dataset size also apply to other soft-core CPU cores. Only in cases with high SNR (e.g., in ML-based processors with more straightforward differentiation between workloads) might our conclusions change: classical and DL methods may display more comparable accuracy if faced with an easy classification problem.

As mentioned earlier, the evaluation presented in this work is limited to embedded soft-core CPUs. Considering more complex processor cores would bring a new set of challenges. Larger CPUs—superscalar, out-of-order, and speculative—entail a higher communication latency, lower operating frequency, and higher area overhead. These factors impact instruction identification accuracy in various ways. On the one hand, a larger area may make the instruction-level leakage stronger. Conversely, the hardware overhead for operating system support or instruction-level parallelism (out-of-order and speculative execution) could increase the noise and reduce the instruction-level leakage. More complex cores might have a lower maximum operating frequency, allowing more sensor samples per CPU clock cycle (i.e., higher quality measurements), but the out-of-order execution could make synchronizing the power traces more difficult. Evaluating the impact of microarchitectural features of larger soft-core CPUs on the instruction-level leakage is, therefore, an interesting avenue for future work.

Our work evaluates instruction-level leakage of soft-core CPUs in isolation. Future research could explore the leakage in the context of a complete system consisting of a soft-core CPU and an accelerator. To further justify the need for mitigations, future work could showcase an attack on longer code sequences, e.g., detecting loops in power traces (with no averaging) and then folding loop executions to obtain averaged traces, or profiling longer open-source code sequences to detect specific code execution. Turning to the countermeasures, Sect. 9 outlines a palette of mitigation techniques that could be implemented in many ways. Evaluating their performance and scalability is important and, as such, merits a study on its own.

Last but not least, the instruction-level leakage evaluation methodology presented in this paper is general and can be used for any CPU microarchitecture to obtain implementation-specific results and conclusions.

## Related Work

In this section, we present the related work, of which the most relevant to ours are power analysis attacks on shared FPGAs and power side-channel disassembly attacks.

### Power Analysis Attacks on Shared FPGAs

Zhao et al. characterized the RO and TDC voltage monitors on an AMD Zynq-7000 SoC and successfully used them in an SPA attack against a (1) collocated RSA cryptomodule and (2) RSA exponentiation running on the ARM processor. The traces they recorded had visibly different amplitude and duration, depending on whether the processed RSA key bit had a binary value of 0 or 1. In a concurrent study, Schnelleberg et al. demonstrated a CPA attack in which, instead of an oscilloscope, they used a TDC sensor collocated with an AES crypto module on an AMD Spartan-6 FPGA [59]. Glamočanin et al. refined the TDC sensor design and ported it on an Amazon EC2 F1 cloud instance (AMD Virtex UltraScale+ FPGA) to showcase a successful CPA attack against a 128-bit AES module. Similarly to Zhao et al., Gravellier et al. targeted AMD Zync-7000 SoC; with correlation power analysis, they recovered the secret key of a bare-metal implementation of Tiny AES and OpenSSL AES [24]. These seminal works showed that physical access is no longer required for power side-channel attacks and that shared FPGAs are vulnerable to power analysis attacks. However, unlike our work, all the attacks mentioned above are statistical-based attacks that depend on thousands or millions of victim execution traces for a successful attack.

Another class of power side-channel attacks on shared FPGAs concerns profiling and reverse engineering another common FPGA workload: neural network accelerators. Given the size of a neuron and the network as a whole, the change in network topology or size can have a considerable (i.e., lasting and distinguishable) impact on the power supply voltage. In a remote attack scenario involving a shared FPGA, it has already been shown that an adversary can infer the activation function, the weights, the number of neurons and layers, the width and depth of convolutional layers, the width of pooling layers, filter sizes, and the stride of convolutional and pooling layers [25–28]. Unlike statistical-based attacks, these profiling attacks require a small number of victim execution traces. However, since the victims are ML accelerators occupying a large portion of the FPGA logic, a good SNR results in easily exploitable side-channel leakage and high attack accuracy. Our work analyzes the leakage of a soft-core CPU, which is a significantly smaller victim than ML accelerators.

In the context of side-channel attacks on ML accelerators, the work of Tian et al. [28] is most relevant to us, as the authors exploit instruction-level leakages of an ML accelerator. The authors use TDC sensor traces to attack a Versatile Tensor Accelerator (VTA) on an AMD Zynq-7000 FPGA. VTA is a generic and customizable deep learning accelerator, which realizes an ML model as a set of VTA instructions and collates them into instruction groups, each containing a mix of LOAD, GEMM, ALU, or STORE instructions. Firstly, Tian et al. have observed that all TDC traces recorded during 25,000 clock cycles (120 MHz clock frequency) for GEMM, ALU-Add, and LOAD-and-STORE unit tests have distinctly different shapes, allowing SPA attacks. Additionally, SPA on the traces recorded during GEMM instructions allows the reverse engineering of the instruction parameters by finding the time interval between adjacent peaks, counting the number of peaks, and measuring the amplitude of the voltage drop in the sensor trace. In our work, we analyze the leakage of a soft-core CPU, considerably smaller than VTA. Moreover, the traces corresponding to the CPU instructions are orders of magnitude shorter in time, and many CPU instructions give extremely similar sensor trace waveforms. Consequently, visual analysis of the traces is not sufficient, and to analyze the instruction-level leakages, we deploy different ML classifiers.

Instead of assuming that the victim is a cryptographic core or a neural network, Gobulukoglu et al. used TDC sensor traces to determine whether a cotenant application is present and what type of application it may be [54]. On an AMD Zynq-7000 FPGA, they deployed one TDC sensor and nine scenarios: one without any cotenant, one power-hungry tenant, and others covering several implementations of AES and PRESENT (a custom IP core, software running on Microblaze, ORCA, and PicoRV *soft-core* processors). They collected 250 sensor traces for each scenario, transformed them into two-dimensional images, and trained the ResNet50 classifier to predict workloads. The reported classification accuracy ranged between 33% and 99%, with an average of around 70%. The lowest classification accuracy was reported between AES and PRESENT running on the same type of soft-core CPU, while the highest was achieved when distinguishing between very different implementations: an AES core and a soft-core CPU. It is worth noting that the soft-core processors were running at 5 MHz, while the sensor was clocked at 100 MHz. In this work, we target a considerably more challenging classification problem; not only is our target soft-core CPU working on a higher frequency, but the information required to determine the instruction the CPU is executing is also contained in a significantly smaller number of sensor samples (shorter trace). Nevertheless, we show that instruction leakage in the power traces is sufficient to achieve an accuracy higher than 80%.

### Power Side-Channel Disassembly Attacks

A body of research covers power side-channel attacks on cryptographic computations, whether they are executed by a CPU or implemented as an ASIC or FPGA circuit. Similarly, researchers investigated whether power side-channel or electromagnetic side-channel emanations can be used to determine the instructions executed by a CPU.

Vermoen et al. were the first to recover the code executed on a Java SmartCard, by correlating the average power traces with a set of templates. Instead of power, Strobel et al. measured the electromagnetic (EM) emanations of an 8-bit PIC16F687 MCU, running at 4 MHz [71]. The first to use ML methods, the authors deployed LDA coupled with the k-NN algorithm and obtained instruction classification accuracy of 96% and 87% for the test and the real codes, respectively. Cristiani et al. focused on the instruction fetch stage of a 14-bit PIC16F15376 MCU operating at a significantly higher frequency than in previous work: 20 MHz [67]. To compensate for a higher CPU frequency, they used a 10 GS/s oscilloscope, averaged 1,000 traces per template to reduce noise, and were the first to record EM side-channel traces at multiple chip locations. Using LDA for dimensionality reduction and a QDA classifier, they reported 95% instruction recognition accuracy. Park et al. targeted an 8-bit ATmega328p MCU (16 MHz, two-stage pipeline) and recorded the power side-channel traces with a 2.5 GS/s oscilloscope [72]. The authors were the first to deploy frequency analysis for disassembly and used CWT to find the differences between the instructions not observable in the time domain. Park et al. then applied Kullback–Leibler (KL) divergence to identify important features, PCA for dimensionality reduction, and a hierarchical classification approach. On the test codes, they reported 99% instruction opcode recognition accuracy. Krishnankutty et al. were the first to find the instruction execution boundaries in a side-channel trace of an MSP430 MCU [69]. Their hierarchical classification based on SVM resulted in 86% opcode recognition accuracy.

Common to the above works is that the victim CPU was running at frequencies orders of magnitude lower than the sampling rate of the oscilloscope for measuring the side-channel traces. On FPGAs, voltage-drop sensors cannot reach the sampling frequencies of an oscilloscope. In our experimental setup, in one case, only four sensor samples were available per one CPU clock cycle, whereas in the other, only one sensor sample was available per CPU clock cycle. Additionally, unlike power disassembly attacks which depend on only one source of power traces, i.e., the oscilloscope, our work leverages multiple remote on-chip sensors to increase the signal. Despite that, we show that ML methods used in power disassembly attacks are not optimal for remote leakage evaluation. We present new DL time-series classifiers that can determine the type of instruction executed with accuracy higher than 80%. Our work not only presents new DL methods beneficial for future power side-channel disassembly attacks, but also shows the need to deploy countermeasures against power disassembly attacks, even in a remote scenario.

## Conclusions

This work analyzes the instruction-level leakages of soft-core CPUs in shared FPGAs. We show that, unlike with ML accelerators, potential attackers cannot rely on SPA alone, as even with significant averaging, the visual leakage of small soft-core CPUs is limited. Instead, to analyze the instruction-level leakages, we compute the classification accuracy using instruction profiling templates. We demonstrate that ML methods from previous power disassembly attacks are insufficient for remote leakage analysis and that evaluators should deploy DL methods: they achieve approximately 10–20% higher accuracy when classifying instructions from power templates. Using DL methods and a worst-case scenario for the evaluator—a breach of physical and logical separation—we achieve a maximum accuracy of 86.42%.

Our analysis demonstrates that as the leakage evaluation scenarios become more realistic for a potential attack, the leakage, and thus the classification accuracy, reduces. Enforcing physical separation and placing the soft-core CPU further away from the on-chip sensors reduces the accuracy significantly, as well as using more realistic templates with the target instruction surrounded by random instructions. Furthermore, we show that most of the instruction-level leakage is constrained to instructions of different types and that the confusion comes from only a few similar instructions: using the top-4 accuracy metric already results in an accuracy above 90% for most of our datasets.

We quantify the impact of averaging on the accuracy and show that the accuracy increases, up to a certain point, as the number of averaged traces increases. We also demonstrate that increasing the number of templates does not significantly increase the accuracy. Furthermore, our analysis shows that a cloud-scale FPGA on the Alveo U200 board has significantly less leakage, as the more prominent and higher quality PDN results in a lower SNR. Finally, we demonstrate that, unlike instruction-level leakages, code sequences exhibit significantly higher leakage and can be classified with an accuracy of 100% even on cloud-scale FPGAs.

Our work can serve as a leakage evaluation methodology for remotely deployed soft-core CPUs. It can also be leveraged for building more advanced power side-channel disassembly attacks.

In conclusion, we demonstrate that even small circuits leak information on shared FPGAs, and that potential attackers can remotely extract that information with a small number of power trace acquisitions. This result highlights the need for deploying appropriate mitigations on soft-core CPUs, in multitenant cloud FPGAs.

## Statements and Declarations

### Data Availability

We make the FPGA designs, associated software, and ML code openly available for the reproducibility of the experiments and the results in this work [34].
